# Regulating NLRP3 Inflammasome–Induced Pyroptosis *via* Nrf2: TBHQ Limits Hyperoxia-Induced Lung Injury in a Mouse Model of Bronchopulmonary Dysplasia

**DOI:** 10.1007/s10753-023-01885-4

**Published:** 2023-08-09

**Authors:** Minrong Wang, Feng Zhang, Xuemei Ning, Chan Wu, Yue Zhou, Zhixian Gou, Yang Fan, Rongrong Duan, Zhongni Li, Chunyan Shao, Liqun Lu

**Affiliations:** 1 Department of Pediatrics, The First Affiliated Hospital of Chengdu Medical College, No. 278, Middle Section of Baoguang Avenue, Xindu District, Chengdu, Sichuan Province 610500 People’s Republic of China; 2 Clinic Medical College, Chengdu Medical College, No. 783 Xindu Avenue, Xindu District, Chengdu, Sichuan Province 610500 People’s Republic of China

**Keywords:** bronchopulmonary dysplasia, Nrf2, ROS, NLRP3, pyroptosis, TBHQ

## Abstract

Nuclear factor e2–related factor 2 (Nrf2) plays a key role in cellular resistance to oxidative stress injury. Oxidative stress injury, caused by Nrf2 imbalance, results in increased pyroptosis, DNA damage, and inflammatory activation, which may lead to the arrest of alveolar development and bronchopulmonary dysplasia (BPD) in premature infants under hyperoxic conditions. We established a BPD mouse model to investigate the effects of tert-butylhydroquinone (TBHQ), an Nrf2 activator, on oxidative stress injury, pyroptosis, NLRP3 inflammasome activation, and alveolar development. TBHQ reduced abnormal cell death in the lung tissue of BPD mice and restored the number and normal structure of the alveoli. TBHQ administration activated the Nrf2/heme oxygenase-1 (HO-1) signaling pathway, resulting in the decrease in the following: reactive oxygen species (ROS), activation of the NOD-like receptor pyrin domain containing 3 (NLRP3) inflammasome, and IL-18 and IL-1β expression and activation, as well as inhibition of pyroptosis. In contrast, after Nrf2 gene knockout in BPD mice, there was more severe oxidative stress injury and cell death in the lungs, there were TUNEL + and NLRP3 + co-positive cells in the alveoli, the pyroptosis was significantly increased, and the development of alveoli was significantly blocked. We demonstrated that TBHQ may promote alveolar development by enhancing Nrf2-induced antioxidation in the lung tissue of BPD mice and that the decrease in the NLRP3 inflammasome and pyroptosis caused by Nrf2 activation may be the underlying mechanism. These results suggest that TBHQ is a promising treatment for lung injury in premature infants with hyperoxia.

## INTRODUCTION

Bronchopulmonary dysplasia (BPD) is a common chronic lung disease that affects premature infants. Clinical studies have shown that the mortality rate in children with moderate to severe BPD can reach approximately 30% [1, 2]. Moreover, BPD is associated with strenuous and prolonged treatment cycles and low cure rates. Furthermore, severe BPD cases often result in long-term complications, such as pulmonary dysfunction and growth retardation, inflicting a heavy toll on families involved, as well as society at large [3]. The pathogenesis of BPD is complex, including oxidative stress, persistent inflammation, increased cell death/apoptosis, protease–antiprotease imbalance, increased collagen fibers, and microvascular changes [4]. With progress in rescue technology in premature infants, the use of pulmonary surfactants and the frequency of mechanical ventilation have increased after birth [5]. Under the continuous stimulation of hyperoxia, an increase in reactive oxygen species (ROS) production and a decrease in antioxidant defense ability lead to oxidative stress injury and alveolar cell death [6–8]. Related studies have indicated that oxidative stress plays an initiating role in BPD [9].

Recent studies have shown that hyperoxia-induced BPD is a potential indicator of persistent oxidative stress in respiratory diseases and that the marker ROS is overexpressed in lung tissue. In addition to activating transcription factors, ROS induces various signaling pathways in cells and regulates different types of cell death [10]. ROS also plays a key role in cell growth and inflammatory responses by continuously activating inflammatory cells and increasing the expression of pro-inflammatory cytokines, leading to respiratory epithelial injury, airway remodeling, and the hindrance of normal alveolar development in children with BPD [11, 12]. As an important transcription factor that protects against oxidative stress–induced injury, nuclear factor e2–related factor 2 (Nrf2) is activated by ROS and plays a key role in maintaining redox homeostasis [13]. Cho *et al.* [14] found that in a hyperoxia-induced BPD model of neonatal mice, Nrf2^+/+^ and Nrf2^−/−^ mice showed different susceptibilities to lung injury and abnormal alveoli from extended periods of hyperoxia (P1–P4) after birth. Furthermore, another study showed that heightened Nrf2 activity can alleviate BPD injury in neonatal mice caused by hyperoxia [15].

BPD pathogenesis is closely related to increased focal death induced by the activation of the NOD-like receptor pyrin domain containing 3 (NLRP3) inflammasome [16]. NLRP3 belongs to the nucleotide-binding oligomerization domain-like receptor (NLR) family, which is a key component of the innate immune system and contains several special domains, such as the central nucleotide domain and oligomerization (NACHT), cysteine protease recruitment (CARD), leucine-rich repeat sequence (lrr), and hot protein (PYD) domains embedded with apoptosis-associated spot-like proteins (ASCs) and Caspase-1 [17, 18]. Once activated, the ASCs and Caspase-1 couple with the NLRP3 inflammatory complex, activating Caspase-1, producing an active p10/p20 tetramer, and inducing the transformation of pro-inflammatory cytokines IL-1β and IL-18 from their immature “precursors” to secretory active forms, activating the downstream inflammatory cascade. The formation of inflammasomes also triggers the process of inflammation-related cell death, also known as “cell scorch death,” which is essentially the death of pro-inflammatory cells [19, 20]. Previous studies [21] have shown that the inhibition of Caspase-1 expression and the reduction in cell death can alleviate hyperoxia-induced lung and brain injuries in neonatal mice. Liao *et al.* [22] found that in the preterm baboon BPD model induced by hyperoxia, targeted deletion of NLRP3 could restrain Caspase-1 activity, significantly downregulate the expression of IL-1β and IL-18, significantly reduce the incidence of cell death, and protect preterm baboons from hyperoxia-induced inflammation, as well as abnormal alveolar simplification.

However, recent studies have found that an imbalance in antioxidant stress injury induced by Nrf2 plays an important role in pyroptosis and participates in disease pathogenesis. Some studies have suggested that mitochondria-derived ROS are key mediators of inflammasome activation in NLRP3 [23]. Related studies have found that the Nrf2 pathway can mitigate oxidative stress injury in diabetic mice, reduce activation of the NLRP3 inflammasome, and improve neuronal death, synaptic damage, and cognitive impairment [24]. Other studies have mentioned that in the rat model of myocardial ischemia/reperfusion injury, inhibiting the expression of NLRP3 and its downstream inflammatory chemokine IL-1β, while upregulating the expression of Nrf2, demonstrates antioxidant and anti-inflammatory effects, as well as significantly reducing cardiac injury [25]. There is evidence that during oxidative stress, ROS responsiveness increases and Nrf2 is activated and enters the nucleus, promoting downstream protective genes, such as heme oxygenase-1 (HO-1) and NADPH quinone dehydrogenase 1, that drive the expression of antioxidant genes, consequently preventing oxidative stress injury and inhibiting cell death [26, 27]. Moreover, NLRP3 inflammasomes mainly exist in immune and inflammatory cells activated by inflammation, and inflammation driven by NLRP3 inflammasomes continues to recruit inflammatory cells, including macrophages, monocytes, and neutrophils, to stimulate the production of ROS, indicating that there is a feedback loop between ROS and NLRP3 inflammasomes. However, the regulatory mechanism remains unclear, warranting further study [28].

In summary, hyperoxia-induced oxidative stress and pyroptosis play important roles in the development of BPD in premature infants. We speculate that Nrf2 may inhibit the activation of the NLRP3 inflammasome and pyroptosis in alveolar cells by regulating oxidative stress injury in BPD; therefore, we provide insights into a promising treatment strategy *via* regulation of the Nrf2/NLRP3/Caspase-1 signaling pathway.

## METHODS

### Animals and BPD Model Generation

Female C57BL/6 mice weighing 20 ~ 25 g and male C57BL/6 mice weighing 25 ~ 30 g, aged 2–3 months, were purchased from Chengdu Dossy Laboratory Animal Co., Ltd. (Sichuan, China). A pair of Nrf2^+/−^ heterozygous C57BL/Nrf2 gene knockout mice were purchased from the Saiye Model Biology Research Center Co., Ltd. (Taicang), and the experiments were approved under the experimental animal quality certificate SYXK 2020–196 (Sichuan). All mice were acclimated to standard cages for 1 week before the experiment commenced and allowed ad libitum access to feed and water. Male and female mice were randomly assigned to cages in a 2:1 ratio. The conditions in the animal facility were maintained at a temperature of 22 ± 2 °C, a relative humidity of 50–60%, and a 12 h/12 h light/dark cycle. Bisex C57BL/6 mouse pups born at E20–E21 were defined as those reaching term delivery and were included in the study. All mice were randomly numbered according to a random number table generated in Excel (Microsoft, Redmond, WA, USA). The random numbers were sorted in increasing order and assigned to different groups. Numerical sample identifiers were used during the experimental procedures and data analysis, and the investigators were blinded to the treatment.

### BPD Sub-model and Grouping

The total number of pups was 252, and all newborn mice were delivered normally and breastfed. Subsequently, they were randomly divided, regardless of sex, with seven newborn mice in each of the following four groups: (1) control group: oxygen concentration maintained at 21% on postnatal days 1–5 (P1–P5); (2) BPD group: receiving 85% oxygen concentration maintenance treatment from P1–P5; (3) BPD + Nrf2^−/−^ group: targeted deletion Nrf2^−/−^ mice receiving 85% oxygen concentration maintenance treatment from P1–P5; and (4) BPD + TBHQ group: receiving an oxygen concentration of 85% with tert-butylhydroquinone (TBHQ) administration (Cat#: HY-100489; MCE, USA) (50 mg/kg) *via* intraperitoneal injection from P1–P5. Thereafter, the normal environment was restored on P7 and P14. The BPD, BPD + Nrf2^−/−^, and BPD + TBHQ groups were stable for 24 h after birth, and the oxygen concentration was maintained at approximately 85%. The oxygen meter was monitored for 24 h, and the quicklime was placed in an oxygen chamber to maintain a dry environment. The control group was exposed to air (oxygen concentration: 21%), and the lactating mice were exchanged once every 24 h to avoid a decrease in feeding ability caused by oxygen poisoning. We followed the “three Rs of animal research: (replace, reduce, and refine)” [29]. An intraperitoneal injection of 4% sodium pentobarbital (200 mg/kg body weight) was administered prior to euthanasia to minimize pain [30]. Each experiment was performed at least six times to improve data quality.

### Histopathology

Six mice were randomly selected from the above groups at P5, P7, and P14. All mice were transcardially perfused with saline after euthanasia, followed by 4% paraformaldehyde. The left lung tissue of the mice was used for paraffin embedding, preparing slices of 5 μm for staining. The right lung tissue was placed in an EP tube, frozen in liquid nitrogen, and kept in a refrigerator at − 80 ℃. Hematoxylin and eosin staining (hue) (Cat# G1121; Solarbio, Beijing, China) was performed using a Leica microscope (DM4000B; Leica, Wetzlar, Germany). Six visual fields were randomly selected from each section. The pathomorphology of the lung tissue was analyzed, and the radial alveolar count (RAC) was measured. A straight line was drawn from the respiratory bronchiole center to the nearest pleura, and the number of alveoli on this line was the RAC value. Image-Pro Plus software (version 6.0) was used to analyze the RAC values.

### Masson’s Staining

The tissue was fixed with 4% formaldehyde solution, sectioned in paraffin, and routinely dewaxed in water, according to the instructions of Masson’s trichrome staining kit (Solarbio, Beijing, China). Finally, a neutral gum seal was applied. Observed under an electron microscope, six visual fields were randomly selected from each section, and images were collected to analyze the pathomorphology of lung tissue. The collagen volume fraction was measured using Image-Pro Plus 6.0 software, similar to before. The staining results showed that the collagen fibers were blue; the muscle fibers, cellulose, and red blood cells were red; and the nucleus was blue-black.

### Terminal Deoxynucleotidyl Transferase dUTP Nick-End Labeling (TUNEL) Assay

Paraffin sections were prepared as previously described. Triton X-100 (0.1%) (Solarbio, China) was used for 5 min, and a 3% H_2_O_2_ sealing solution was prepared and used for washing three times at 25 ± 2 °C for 10 min. A TUNEL apoptosis detection kit and a core package (KeyGEN, Jiangsu, China) were used for staining. Finally, DAB working solution color treatment was applied, followed by three consecutive washes with PBS, hematoxylin or methyl green re-staining, and sealing with neutral gum. The Servicebio CF488 Tunel Cell Apoptosis Detection Kit was used for co-labeling experiments. For the experimental methods, refer to the reagent specification.

### Immunofluorescence

Paraffin sections were prepared as previously described. Sections were washed thrice with PBS; thereafter, 0.1% Triton X-100 (Solarbio, China) was allowed to permeate for 5 min at room temperature, followed by three consecutive washes with PBS. Then, dripping of an appropriate amount of 3% BSA (Solarbio, China) was applied, with sealing at room temperature for 10 min. Subsequently, an appropriate amount of target-first antibody was added: rabbit anti-Nrf2 polyclonal antibody (Abcam; 1:100; ab62352), rabbit anti-NLRP3 polyclonal antibody (Abcam; 1:100; ab270449), and mouse anti-Caspase-1 polyclonal antibody (Proteintech; 1:100; 22,915–1-AP) prior to 12 h incubation at 4 ℃. Thereafter, the samples were incubated with goat anti-rabbit IgG antibody (Proteintech; 1:100; SA00009-2) labeled with Cy3 and goat anti-mouse IgG antibody (Proteintech; 1:300; 68,132–1-lg) labeled with FITC and incubated at 37 ℃ without light for 1 h. During the final 5 min of incubation, the sections were sealed with Antifade Mounting Medium (Beyotime, China). Images were acquired using fluorescence microscopy, and ImageJ software (Rasband, W.S., ImageJ; National Institutes of Health, Bethesda, MD, USA) was employed for the fluorescence intensity analysis.

### Dihydroethidium

ROS levels in the lung tissue were determined with dihydroethidium dye (Beyotime, 1:10000, S0063) according to the manufacturer’s instructions. Frozen sections (5 μm) were prepared, followed by the addition of an appropriate dilution of dihydroethidium and incubation at room temperature for 15 min. The tablets were then sealed with Antifade Mounting Medium (Solarbio, Beijing, China). Images were collected and analyzed using an electron microscope at an excitation wavelength of 460–610 nm.

### Western Blot Analysis

Proteins were extracted using a BCA protein concentration assay kit (Solarbio, Beijing, China). An SDS-PAGE gel was prepared for electrophoresis and then transferred onto a PVDF membrane at 250 mA for 1.5 h, followed by incubation with 5% skim milk powder for 45 min. Subsequently, the following primary antibodies were incubated at 4 °C: rabbit anti-Nrf2 polyclonal antibody (Abcam; 1:1000; ab62352), rabbit anti-NLRP3 polyclonal antibody (Abcam; 1:1000; ab270449), mouse anti-Caspase-1 polyclonal antibody (Proteintech; 1:1000; 22915–1-AP), rabbit anti-IL-1β polyclonal antibody (Proteintech; 1:1000; 16806–1-AP), rabbit anti-IL-18 polyclonal antibody (Proteintech; 1:1000; 10663–1-AP), mouse anti-HO-1 monoclonal antibody (Abcam; 1:1000; ab305290), and mouse anti-β-Actin monoclonal antibody (Proteintech; 1:5000; 81115–1-RR). The following secondary antibodies were incubated at 25 ± 2 °C the next day for 2 h: goat anti-rabbit IgG or goat anti-mouse IgG (Santa Cruz Biotechnology, 1:10000 dilution). An ECL (Biosharp, Beijing, China) luminescent working solution was prepared, and the membrane was uniformly incubated at 12 h in the dark for 2–3 min. Images were collected and analyzed using a gel imaging system.

### Statistical Analysis

Data are presented as the mean ± standard error of the mean (SEM). On account of the small sample size of the animal experiments, no outlier tests were conducted. The Shapiro–Wilk test was used to assess the normality of the experimental data. An unpaired *t*-test was used to compare the means of the two groups. Multiple groups were compared using analysis of variance (ANOVA). All graphs were prepared using Prism 8.02 software (RRID: SCR_002798; GraphPad, San Diego, CA, USA). The Statistical Package for the Social Sciences (SPSS) 25.0 (RRID: SCR_002865; IBM, Armonk, NY, USA) was used for the statistical analyses. Statistical significance was set at *P* < 0.05.

## RESULTS

### Establishment of a Neonatal Mouse Model of Hyperoxia-Induced BPD

**Fig. 1:**
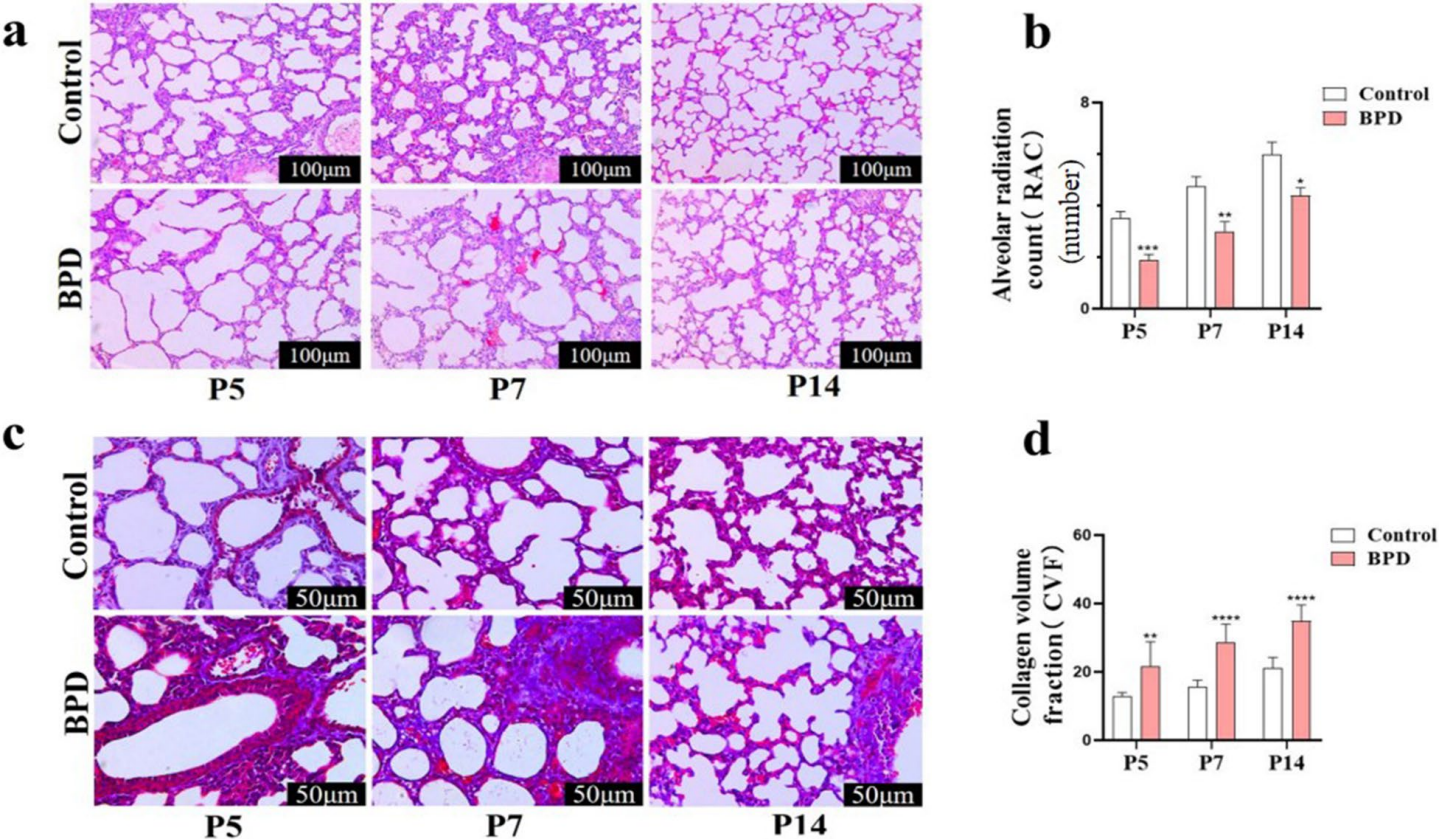
The hyperoxia-induced BPD neonatal mouse model was established. **a**, **b** The pathological changes of lung tissue at P5, P7, and P14 in the control and BPD groups were evaluated with H&E staining, and RAC statistical analysis was performed. Data are expressed as the mean ± SEM for *n* = 6 mice/group (one asterisk denotes *P* < 0.05; two asterisks denote *P* < 0.01; three asterisks denote *P* < 0.001). Scale bars = 100 μm. **c**, **d** The changes in lung collagen fibers at P5, P7, and P14 in the control and BPD groups were evaluated by Masson’s staining, and CVF quantitative analysis was performed. Data are expressed as mean ± SEM for *n* = 6 mice/group (one asterisk denotes *P* < 0.05; three asterisks denote *P* < 0.001). Scale bars = 50 μm.

First, we collected the lung tissues of newborn P5, P7, and P14 mice with BPD and observed the pathological morphology using H&E staining (Fig. 1a). Our data demonstrated that, compared with the corresponding parameters in the control group, the newborn mice in the BPD group had lung tissue structure disorder, alveolar wall rupture and destruction, a significantly reduced number of alveoli fused into large alveoli, and alveolar interstitial edema and widening. The RAC values indicated that the number of alveoli at P5, P7, and P14 in the BPD group was significantly lower than that in the control group (Fig. 1b) (*P* < 0.001 at P5, *P* < 0.01 at P7, *P* < 0.05 at P14). Masson’s trichrome staining was used to determine the presence of collagen fibers and alveolar septum thickening in the lung tissues of hyperoxia-induced BPD neonatal mice (Fig. 1c). The collagen volume fraction (CVF) value showed that the volume fraction of collagen in the lung structure of neonatal mice in the BPD group at P5, P7, and P14 was significantly higher than that in the control group (Fig. 1d) (*P* < 0.01 at P5, *P* < 0.0001 at P7, *P* < 0.0001 at P14).

### Nrf2 Oxygen Factor, Chemical Stress Antioxidant Factor, and Oxidative Stress Balance in the BPD Group

**Fig. 2:**
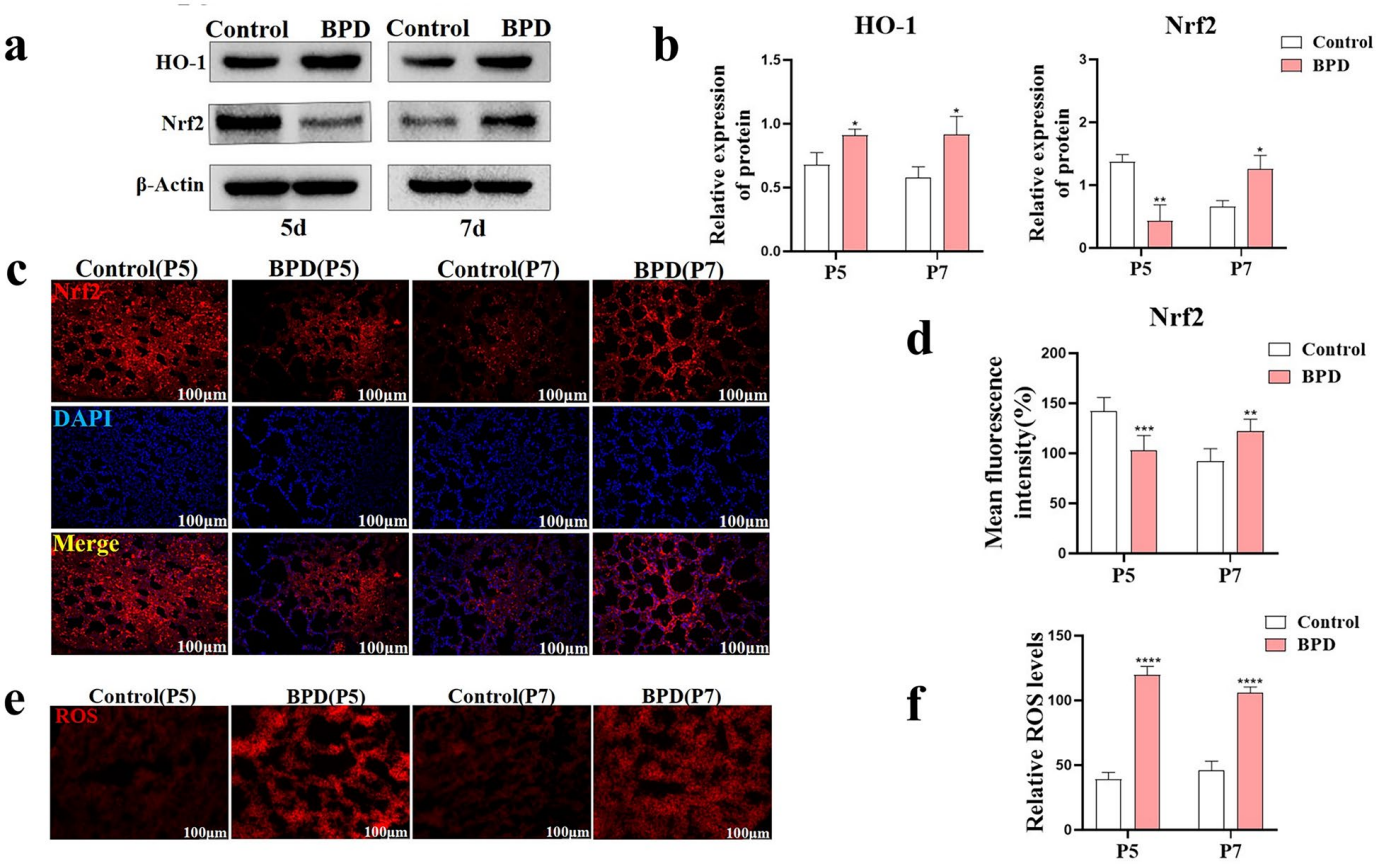
Nrf2 antioxidant factor and oxidative stress imbalance in the BPD group. **a**, **b** The expression of Nrf2 and HO-1 after hyperoxia treatment was detected by western blot with β-actin as the reference, and quantitative analysis was performed. Data are expressed as the mean ± SEM for *n* = 6 mice/group (one asterisk denotes *P* < 0.05; two asterisks denote *P* < 0.01). **c**, **d** Immunofluorescence was used to detect the expression of Nrf2 (Cy3 red) in lung tissues of the control and BPD groups at P5 and P7, and the difference in average fluorescence intensity was analyzed. The nuclei were stained with DAPI (blue). Data are expressed as the mean ± SEM for *n* = 6 mice/group (two asterisks denote *P* < 0.01; three asterisks denote *P* < 0.001). Scale bars = 100 μm. **e**, **f** DHE-ROS was used to detect and quantify ROS content in lung tissue of the control and BPD groups at P5 and P7. Data are expressed as the mean ± SEM for *n* = 6 mice/group (two asterisks denote *P* < 0.01; three asterisks denote *P* < 0.001; four asterisks denote* P* < 0.0001). Scale bars = 100 μm.

By comparing the expression of Nrf2 and HO-1 in the lung tissues of the control group and the BPD group using western blotting, we found that at P5, the Nrf2 expression in the BPD group was significantly downregulated, but the expression of HO-1 was increased (all *P* < 0.05) (Fig. 2a, b), suggesting an imbalance in the antioxidant stress system. Compared with the corresponding expression levels in the control group, two days after the cessation of hyperoxia stimulation, the expression levels of Nrf2 and HO-1 in the lung tissue of the BPD group at P7 increased (all *P* < 0.05) (Fig. 2a, b), and the feedback of antioxidant factors was upregulated. Simultaneously, fluorescence staining of Nrf2 in the lung tissues of the control group and the BPD group showed that the expression of Nrf2 was relatively decreased at P5 (*P* < 0.001) and increased at P7 (*P* < 0.01) (Fig. 2c, d) in the BPD group. The DHE-ROS test results showed that, compared with the ROS level in the control group, the ROS level in the lung tissue of the BPD group was significantly increased at P5 (*P* < 0.001) (Fig. 2e, f).

### Hyperoxia-Induced BPD Mice Showed Increased Pyroptosis Caused by the NLRP3 Inflammasome

**Fig. 3:**
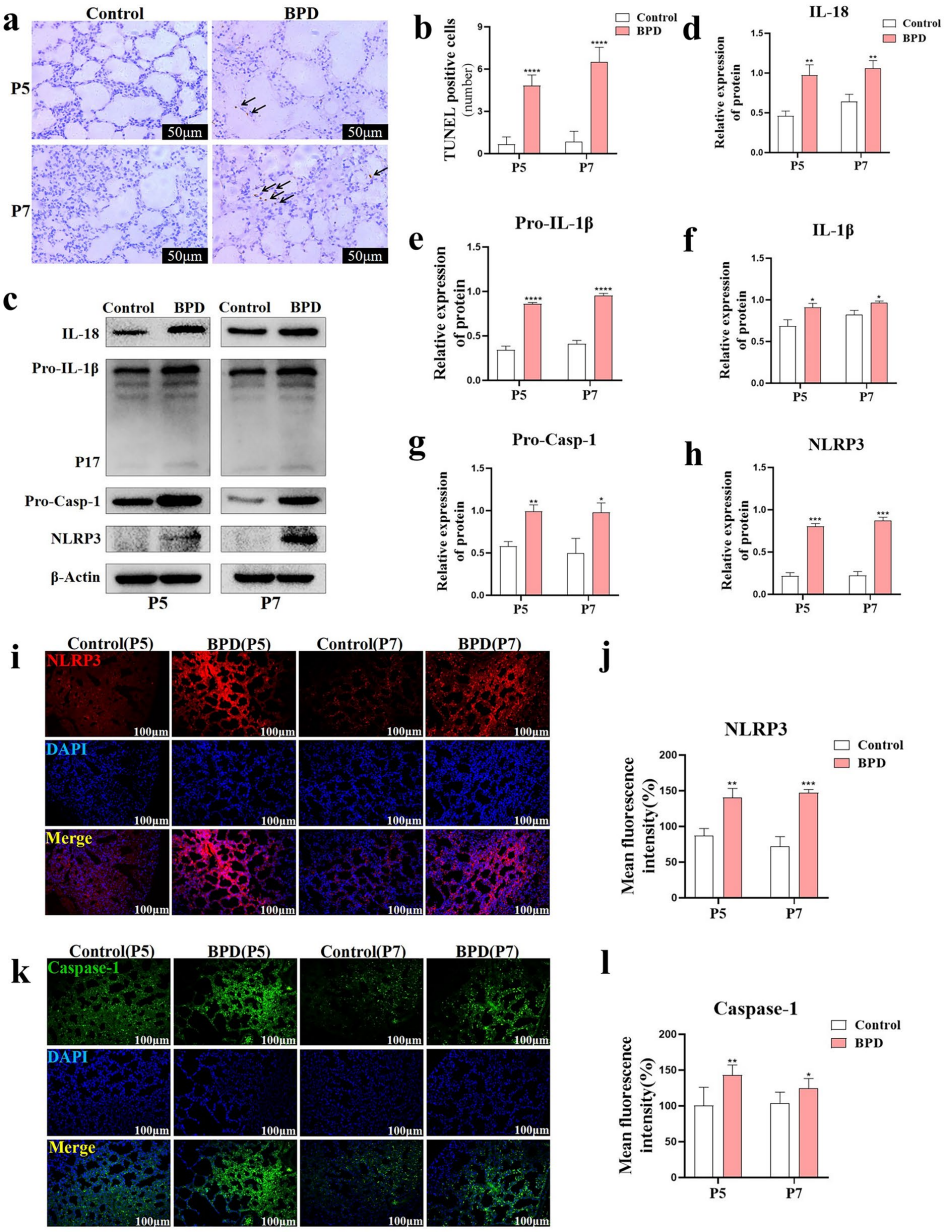
Hyperoxia-induced BPD mice have increased pyroptosis induced by the NLRP3 inflammasome. **a**, **b** TUNEL *in situ* staining was used to observe and analyze the effect of hyperoxia on TUNEL-positive cells in the lung tissue of neonatal mice. Data are expressed as the mean ± SEM for *n* = 6 mice/group (four asterisks denote* P* < 0.0001). **c**–**h** Western blotting was used to detect NLRP3, Caspase-1, IL-18, and IL-1β in the lung tissues of the control and BPD groups at P5 and P7. Data are expressed as the mean ± SEM for *n* = 6 mice/group (one asterisk denotes *P* < 0.05; two asterisks denote *P* < 0.01; three asterisks denote* P* < 0.001; four asterisks denote* P* < 0.0001). **i**–**l** *In situ* immunofluorescence detection of NLRP3 and Caspase-1, and the average fluorescence intensity. Data are expressed as the mean ± SEM for *n* = 6 mice/group (one asterisk denotes* P* < 0.05; two asterisks denote* P* < 0.01; three asterisks denote* P* < 0.001). Scale bars = 100 μm.

Compared with the TUNEL-positive cells in the control group, the number of TUNEL-positive cells in the lung tissue of neonatal mice was significantly increased in the BPD group (all *P* < 0.0001) (Fig. 3a, b). Protein blotting showed that the expression levels of NLRP3 (all *P* < 0.001), Caspase-1 (*P* < 0.01 at P5 and *P* < 0.05 at P7), IL-18 (all *P* < 0.01), Pro-IL-1β (all *P* < 0.0001), and IL-1β (all *P* < 0.01) increased in P5 and P7 in the BPD group (Fig. 3c–h). *In situ* fluorescence staining of lung tissue showed that the expression levels of NLRP3 (*P* < 0.01 at P5 and *P* < 0.001 at P7) and Caspase-1 (*P* < 0.01 at P5 and *P* < 0.05 at P7) in the BPD group increased significantly at P5 and P7 (Fig. 3i–l), consistent with our western blot results. These results demonstrated significant pyroptosis in the BPD group.

### Effect of TBHQ on Lung Development in the BPD Group

**Fig. 4:**
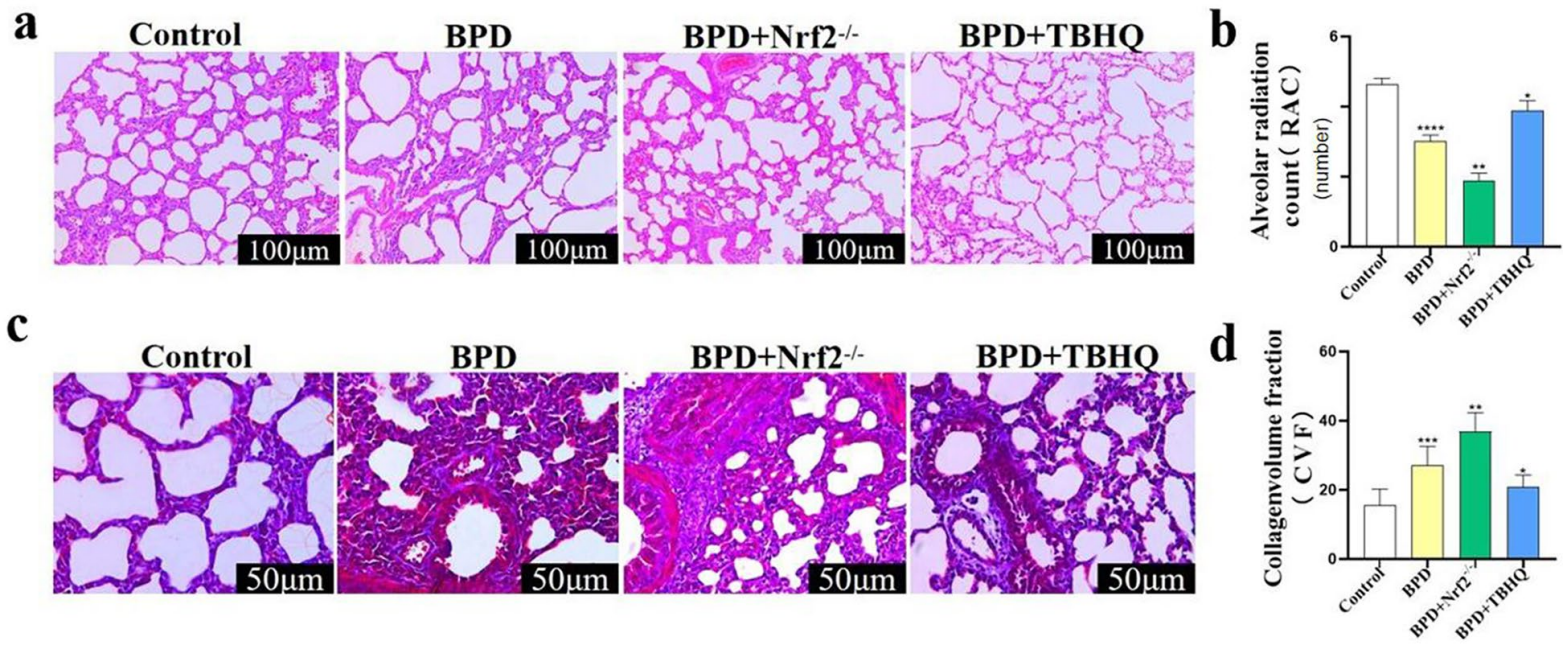
Effect of TBHQ on lung development in the BPD group. **a**, **b** The RAC values of the lung tissue at P7 in the control, BPD, BPD + Nrf2^−/−^, and BPD + TBHQ groups were evaluated by pathological staining. Data are expressed as the mean ± SEM for *n* = 6 mice/group (two asterisks denote* P* < 0.01; four asterisks denote* P* < 0.0001). Scale bars = 100 μm. **c**, **d** The changes in collagen fibers in the lung tissue at P7 in the control, BPD, BPD + Nrf2^−/−^, and BPD + TBHQ groups were evaluated by Masson’s staining, and CVF quantitative analysis was performed. Data are expressed as the mean ± SEM for *n* = 6 mice/group (one asterisk denotes* P* < 0.05; two asterisks denote* P* < 0.01; three asterisks denote* P* < 0.001). *P represents the statistical data values of the control and BPD groups, the BPD and BPD + Nrf2^−/−^ groups, and the BPD and BPD + TBHQ groups in turn. Scale bars = 50 μm.

H&E staining of lung tissues of newborn mice at P7 showed that, compared with the BPD group, the simplification and disorder of lung structure in the BPD + Nrf2^−/−^ group was more serious, the number of alveoli was reduced (*P* < 0.01), the RAC value increased after TBHQ treatment (Fig. 4a, b), and the alveolar structure was more regular. Masson’s staining of the lung tissue at P7 showed that the CVF value of the BPD + Nrf2^−/−^ group was significantly higher than that of the BPD group (*P* < 0.01), and thickening of the collagen fibers and alveolar septa in the BPD + TBHQ group was significantly decreased (*P* < 0.05) (Fig. 4c, d).

### Effect of TBHQ on Oxidative Stress in Lung Tissue of the BPD Group

**Fig. 5:**
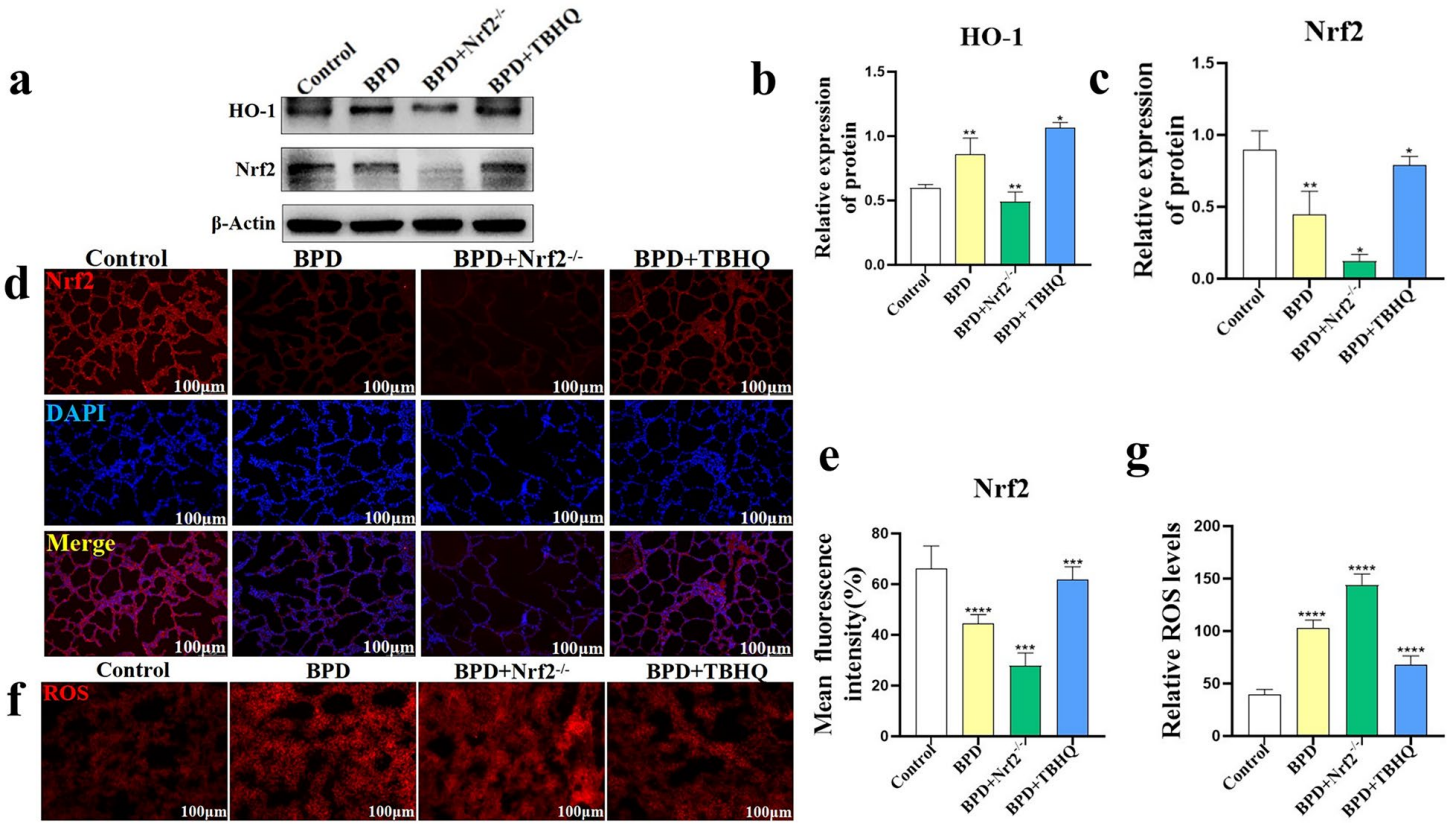
Effect of TBHQ on oxidative stress in the lung tissue of the BPD group. **a**–**c** Immunoblotting was used to detect the protein expression of Nrf2 and HO-1 in the lung tissues of the control, BPD, BPD + Nrf2^−/−^, and BPD + TBHQ groups at P5. Data are expressed as the mean ± SEM for *n* = 6 mice/group (one asterisk denotes* P* < 0.05; two asterisks denote* P* < 0.01). **d**, **e** The average immunofluorescence intensity of Nrf2 in the lung tissues of the control, BPD, BPD + Nrf2^−/−^, and BPD + TBHQ groups was compared. Data are expressed as the mean ± SEM for *n* = 6 mice/group (three asterisks denote* P* < 0.001; four asterisks denote* P* < 0.0001). Scale bars = 100 μm. **f**, **g** DHE-ROS was used to detect and quantify ROS content in the lung tissues of the control, BPD, BPD + Nrf2^−/−^, and BPD + TBHQ groups. Data are expressed as the mean ± SEM for *n* = 6 mice/group (four asterisks denote* P* < 0.0001). *P represents the statistical data values of the control and BPD groups, the BPD and BPD + Nrf2^−/−^ groups, and the BPD and BPD + TBHQ groups in turn. Scale bars = 100 μm.

Western blotting results showed that, compared to the BPD group, the expression levels of HO-1 (*P* < 0.05) and Nrf2 (*P* < 0.05) in the lung tissue of newborn mice in the BPD + TBHQ group increased significantly at P5, whereas the expression of HO-1 (*P* < 0.01) and Nrf2 (*P* < 0.05) protein in the BPD + Nrf2^−/−^ group was significantly inhibited (Fig. 5a–c). Furthermore, Nrf2 fluorescence staining analysis showed that, compared with the BPD group, Nrf2 expression in the lung tissue of the BPD + TBHQ group was significantly increased after TBHQ activated Nrf2 (*P* < 0.001), and Nrf2 levels in the BPD + Nrf2^−/−^ group were significantly inhibited (*P* < 0.001) (Fig. 5d, e). The DHE-ROS test results of newborn mice in the control group, the BPD group, the BPD + Nrf2^−/−^ group, and the BPD + TBHQ group showed that the ROS level in the lung tissue of the BPD + TBHQ group was significantly lower than that in the BPD group (*P* < 0.0001), whereas the ROS level of the BPD + Nrf2^−/−^ group was significantly higher (*P* < 0.0001) (Fig. 5f, g).

### Effect of TBHQ on Pyroptosis in Lung Tissue of Newborn Mice in the BPD Group

**Fig. 6:**
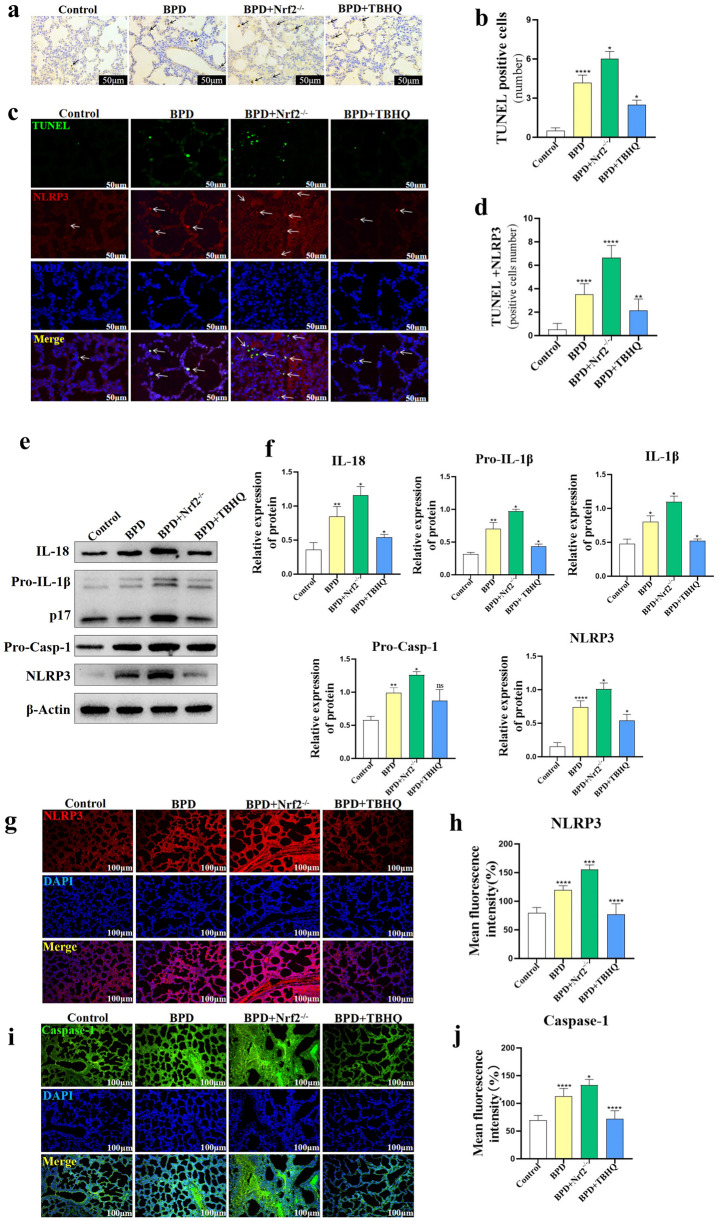
Effect of TBHQ on pyroptosis in lung tissue of newborn mice in the BPD group. **a**, **b** TUNEL staining was used to analyze the degree of cell death in lung tissue of newborn mice at P5 in the control, BPD, BPD + Nrf2^−/−^, and BPD + TBHQ groups. Data are expressed as mean ± SEM for *n* = 6 mice/group (one asterisk denotes* P* < 0.05; two asterisks denote* P* < 0.01; four asterisks denote* P* < 0.0001). Scale bars = 50 μm. **c**, **d** TUNEL + and NLRP3 + co-staining were used to analyze the number of TUNEL + and NLRP3 + co-positive cells in lung tissue of neonatal mice in the control group, the BPD group, the BPD + Nrf2^−/−^ group, and the BPD + TBHQ group at P5. Data are expressed as mean ± SEM of *n* = 6 mice/group (two asterisks denote* P* < 0.01; four asterisks denote* P* < 0.0001). Scale bar = 50 μm. **e**, **f** Immunoblotting method was used to detect NLRP3, Caspase-1, IL-18, and IL-1β in lung tissue of the control group, the BPD group, the BPD + Nrf2^−/−^ group, and the BPD + TBHQ group Protein expression and activation level. Data are expressed as the mean ± SEM for *n* = 6 mice/group (one asterisk denotes* P* < 0.05; two asterisks denote* P* < 0.01; four asterisks denote* P* < 0.0001). **g**–**j** The protein contents of NLRP3 and Caspase-1 in lung tissues of the control group, the BPD group, the BPD + Nrf2^−/−^ group, and the BPD + TBHQ group were determined by immunofluorescence staining. Data are expressed as mean ± SEM for *n* = 6 mice/group (one asterisk denotes* P* < 0.05; three asterisks denote* P* < 0.001; four asterisks denote* P* < 0.0001). *P represents the statistical data values of the control and BPD groups, the BPD and BPD + Nrf2^−/−^ groups, and the BPD and BPD + TBHQ groups in turn. Scale bars = 100 μm.

*In situ* TUNEL staining showed that the number of TUNEL-positive cells in the lung tissue of newborn mice in the BPD + TBHQ group decreased significantly at P5 (*P* < 0.05), whereas the number of apoptotic cells in the BPD + Nrf2^−/−^ group increased significantly (*P* < 0.05) (Fig. 6a, b). TUNEL and NLRP3 co-staining showed that NLRP3 activation–induced pyrodeath of alveolar cells in the BPD group was significantly increased (*P* < 0.0001), and the number of pyroptosis-positive cells in the TBHQ group was significantly lower than that in the BPD group (*P* < 0.01); conversely, the Nrf − / − group had the opposite result, and the number of pyroptosis-positive cells was significantly increased at P5 (*P* < 0.0001) (Fig. 6c, d). The expression levels of NLRP3 (*P* < 0.05), Caspase-1, IL-18 (*P* < 0.05), Pro-IL-1β (*P* < 0.05), and IL-1β (*P* < 0.05) in the lung tissue of the BPD + TBHQ group were significantly inhibited, as shown by western blot. The levels of NLRP3 (*P* < 0.05), Caspase-1 (*P* < 0.05), IL-18 (*P* < 0.05), Pro-IL-1β (*P* < 0.05), and IL-1β (*P* < 0.05) increased significantly after Nrf2 knockout in neonatal mice (Fig. 6e, f). The results of fluorescence staining showed that the expression levels of NLRP3 (*P* < 0.0001) and Caspase-1 (*P* < 0.0001) in lung tissue of neonatal mice at P5 in the BPD + TBHQ group decreased, while the mean fluorescence intensity of NLRP3 (*P* < 0.001) and Caspase-1 (*P* < 0.05) in the BPD + Nrf2^−/−^ group increased (Fig. 6g–j).

## DISCUSSION

With continuous improvements in medical technology, the survival and perinatal survival rates of premature infants have increased significantly [31]. As a common chronic lung disease in premature infants, the high severity of BPD causes high neonatal morbidity, in-hospital mortality, and frequent use of continuous respiratory support after discharge [32]. Statistics show that babies with a birth weight of less than 1000 g have a 70% chance of developing BPD, while babies between 1000 and 1500 g have a 29.3% chance [33]. Simultaneously, the incidence of late-related complications is also increasing, such as persistent respiratory symptoms, abnormal pulmonary function, pulmonary hypertension, and neurodevelopmental disorders [34]. Prenatal factors such as intrauterine growth restriction, corticosteroid deficiency, chorioamnionitis, preterm birth, and low birth weight, as well as postpartum factors such as hyperoxia, mechanical ventilation, patent ductus arteriosus, systemic inflammatory reaction, and infection, are all risk factors for BPD [33, 35]. Currently, it is believed that acute lung injury after exposure to hyperoxia plays an important role in promoting the development of BPD in premature infants. Long-term oxygen therapy in premature infants leads to an increase in ROS, which leads to a continuous state of oxidative stress enhancement and has an irreversible effect on the alveoli during the critical period of alveolar development (23–32 weeks of age in humans) [36, 37].

Our study also found that hyperoxia-induced BPD in neonatal mouse models at P5, P7, and P14 showed lung tissue structure disorder, alveolar wall rupture, decreased RAC values, stagnant alveolar development, and increased CVF values, suggesting thickening of collagen fibers and the alveolar septum. Moreover, we detected that the level of ROS in the BPD group was significantly higher than that in the control group at P5 and P7, indicating the existence of oxidative stress injury. As an important antioxidant transcription factor [38], Nrf2 promotes the expression of many antioxidant enzymes, such as HO-1, glutathione, and thioredoxin [39], and therefore, plays a key role in antioxidant stress injury. Previous studies have reported that the activation of Nrf2 can regulate the activity of antioxidant response elements, enhance the expression of downstream antioxidant enzymes, and enhance antioxidant capacity, thereby simplifying the alveolar structure in BPD mice [40]. Moreover, Nrf2-deficient mice show increased susceptibility to and severity of various respiratory diseases, including BPD [6]. Previous studies have shown that in neonatal mice exposed to hyperoxia, Nrf2 deficiency aggravates lung injury, while Nrf2 activation increases the survival rate of mice, indicating that Nrf2 plays an important role in resisting oxidative stress injury [41, 42]. We also found that the feedback of Nrf2 in the lung tissue of the BPD group decreased during the P5 hyperoxia maintenance period, suggesting that the antioxidant capacity of the BPD group decreased; however, the level of HO-1, one of the downstream factors, increased unexpectedly, indicating that there was an imbalance of oxidative stress homeostasis in the BPD group, possibly because of the upregulation of HO-1 protection caused by other pathways. After interrupting hyperoxic stimulation for two days, the expression levels of Nrf2 and HO-1 in the lung tissue of the BPD group increased at P7, which may indicate an improvement in antioxidant capacity after oxidative stress injury. In addition to hyperoxia, the antioxidant capacity of organisms increases with feedback, which has also been confirmed in previous studies [43, 44].

Presently, it is believed that oxidative stress injury may lead to adverse effects, such as increased pyroptosis, DNA damage, and inflammatory activation of tissues and cells; however, its specific mechanism in BPD is unknown. The current study indicated an oxidative stress imbalance as well as increased pyroptosis in the BPD group. After hyperoxia stimulation, the amount of cell death in the lung tissue of neonatal mice in the BPD group increased significantly at P5 and P7, as well as NLRP3, Caspase-1, IL-18, Pro-IL-1β, and IL-1β, which are markers of cell pyrolysis and activation of the NLRP3 inflammasome. Furthermore, a previous report showed that in neonatal mice, hyperoxia exposure and overexpansion during mechanical ventilation were found to help activate the NLRP3 inflammasome, significantly increase the expression and activation of Caspase-1 and IL-1β proteins, and induce pyroptosis in the lung tissue of BPD mice. Meanwhile, the decrease in the NLRP3 inflammasome resulted in a significantly decreased inflammatory response, and succeeding NLRP3 gene knockout, the alveoli increased, and the BPD phenotype was ameliorated [22]. Although previous studies have found that Nrf2 plays an important role in regulating oxidative stress injury, inflammation, immunity, apoptosis/scorch death, and carcinogenesis, the specific mechanisms involving Nrf2, through which hyperoxia activates the NLRP3 inflammasome, require further study [39, 45]. Dhar *et al.* [46] found that in a mouse model of LPS-induced acute lung injury, Nrf2 inhibited the activation of inflammasomes and pyroptosis in macrophages and reduced lung injury by regulating the ROS/Nrf2/NLRP3 pathway. Moreover, some studies have found that regulating the Nrf2/NLRP3 signaling pathway may alleviate LPS-induced acute lung injury *via* lipopolysaccharide [47]. It has been suggested that Nrf2 and its downstream target NLRP3 play an important role in the regulation of pyroptosis, indicating a correlation between oxidative stress and pyroptosis. Additionally, several reports have indicated that ventilator-induced lung injury may be alleviated by regulating the Nrf2/NLRP3 signaling pathway [48]. Other studies have shown that the activation of Nrf2 can inhibit the assembly and activation of the NLRP3 inflammasome, hence significantly decreasing the expression of downstream effectors, such as Caspase-1, ASC, and IL-18 [49, 50]. This suggests that the Nrf2/NLRP3 pathway may be a robust therapeutic target for hyperoxia-induced lung injury. Therefore, combined with the results of this study, we speculate that the excessive production of ROS in the lung tissue of premature infants after hyperoxia exposure destroys the balance between oxidation and antioxidation and triggers oxidative stress. Additionally, an imbalance in antioxidant factors represented by Nrf2 may be one of the main mechanisms leading to pyroptosis, lung inflammation, and injury.

Hence, the targeted activation of Nrf2 to resist oxidative stress injury, reduce lung epithelial cell death, and promote normal alveoli may be a potential strategy for treating BPD. TBHQ is a widely used food preservative with potent antioxidant effects, oil solubility, low toxicity, safety, and antibacterial activity [51]. Studies have shown that TBHQ plays a significant antioxidant role in various diseases, mainly by modifying the mercaptan group of cysteine on Keap1 to effectively activate Nrf2 [52]. In previous studies, TBHQ has been shown to play a protective role by inhibiting oxidative stress injury and reducing abnormal cell death [53, 54]. A recent study reported that TBHQ reduces the expression of ROS and increases the expression of oxidative proteases, such as HO-1, through antioxidant stress, antagonizing abnormal cell death, and reducing neurotoxicity [55]. In this study, we found that the disturbance of alveolar development in BPD mice improved after TBHQ treatment. In addition, the RAC values increased and CVF values decreased at P7, which enhanced the lung development of BPD mice. In contrast, alveolar development was significantly hindered following the knockout of Nrf2. Furthermore, TBHQ decreased the ROS content, increased the expression of Nrf2, and increased the level of HO-1 protein in the lung tissue of BPD mice. TBHQ can also inhibit the expression and activation of cell death–related proteins, i.e., NLRP3, Caspase-1, IL-18, Pro-IL-1β, and IL-1β, and there was a significant decrease in TUNEL + and NLRP3 + co-positive cells in the alveoli. Compared with the TBHQ-treated group, our results demonstrated an opposite trend in the lung tissue of BPD mice, with targeted deletion of Nrf2 oxidative stress markers and cell death–related proteins.

## CONCLUSION

**Fig. 7:**
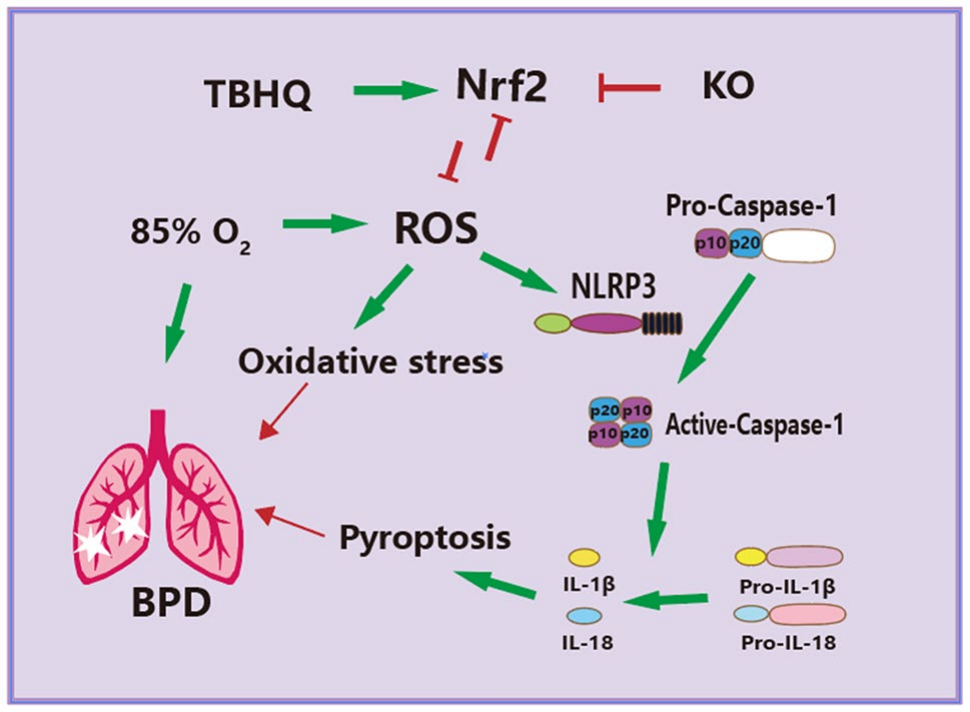
In the model of hyperoxia (85% O_2_)-induced BPD in newborn mice, TBHQ can reduce the production of ROS, oxidative stress injury, and the degree of pyroptosis in lung tissue. This is achieved by regulating the Nrf2/NLRP3/Caspase-1 signaling pathway. The expression of the antioxidant protein Nrf2 was unbalanced in newborn mice under hyperoxia stimulation. Simultaneously, the NLRP3 protein was overregulated in the lung tissue, which promoted the activation and expression of Caspase-1, resulting in significant upregulation of pyroptosis proteins and inflammatory factors (IL-18 and IL-1β). Post-administration of the Nrf2 agonist drug TBHQ, pyroptosis in the lung tissue of BPD was reduced, and the number and normal structure of the alveoli was restored. Furthermore, mice with targeted knockout (KO) of the Nrf2 gene obtained opposing results.

TBHQ enhanced the level of antioxidation in the lung tissue of BPD mice by activating Nrf2 and inhibiting the activation of the NLRP3 inflammasome and pyroptosis, in addition to promoting alveolar formation and maturation—the mechanism of which is summarized in Fig. 7. Our findings suggest that TBHQ is a potential drug for the prevention of BPD in premature infants with hyperoxia. Additionally, the current study indicates that during hyperoxia treatment in premature infants, any approach or drug that can activate Nrf2 and inhibit the NLRP3 inflammasome may serve as an effective therapeutic strategy for BPD.

## Data Availability

The datasets used and/or analyzed during the current study are available from the corresponding author on reasonable request.

## Abbreviations

ANOVA: Analysis of variance
BPD: Bronchopulmonary dysplasia
CVF: Collagen volume fraction
H&E: Hematoxylin and eosin
HO-1: Heme oxygenase-1
IL-18: Interleukin 18
IL-1β: Interleukin 1β
NLRP3: NOD-like receptor pyrin domain containing 3
Nrf2: Nuclear factor e2–related factor 2
P: Postnatal time point
RAC: Radial alveolar count
ROS: Reactive oxygen species
SDS-PAGE: Sodium dodecyl sulfate–polyacrylamide gel electrophoresis
SEM: Standard error of the mean
TBHQ: Tert-butylhydroquinone
TUNEL: Terminal deoxynucleotidyl transferase dUTP nick-end labeling
